# A Substance-Based Medical Device for Managing Hemorrhoidal Disease: Output from a Cross-Sectional Survey

**DOI:** 10.3390/jcm14176069

**Published:** 2025-08-27

**Authors:** Roberto Cioeta, Paola Muti, Marta Rigoni, Roberta La Salvia, Elena Gabriele, Andrea Cossu, Emiliano Giovagnoni

**Affiliations:** 1 Aboca SpA, 52037 Sansepolcro, Italy; rlasalvia@aboca.it (R.L.S.); egabriele@aboca.it (E.G.); acossu@aboca.it (A.C.);; 2 Department of Biomedical, Surgical and Dental Sciences, University of Milan, 20122 Milan, Italy; paola.muti@unimi.it (P.M.); marta.rigoni@unimi.it (M.R.); 3 Istituto di Ricovero e Cura a Carattere Scientifico I.R.C.C.S. Multimedica, 20099 Milan, Italy

**Keywords:** hemorrhoidal disease, real-word evidence, RWE, natural substance-based medical device, safety, effectiveness

## Abstract

**Background:** Hemorrhoidal disease (HD) is a common anorectal condition characterized by symptoms such as bleeding, pain, discomfort and itching. While often underdiagnosed due to patient reluctance to seek care, HD significantly impacts quality of life (QoL). Conservative treatments are preferred for low-grade HD, with increasing interest in natural substance-based therapies. **Materials and Methods:** A large-scale survey was conducted using a digital platform to collect real-world data (RWD) from patients, pharmacists and physicians. The research assessed perceived effectiveness, safety, tolerability, quality of life (QoL) and usage patterns of NeoFitoroid BioOintment. Quantitative analysis was also performed using a global score (GS) based on Likert scale ratings and their distribution. **Results:** A total of 2618 participants were included. A strong concordance across the three participating cohorts in the answers provided for all items of the questionnaire was observed. The descriptive analysis revealed high grades of effectiveness, safety and tolerability. Indeed, over 90% of respondents rated the product as “*good*” or “*excellent*” in terms of effectiveness and safety. **Conclusions:** These findings underscore the treatment’s effectiveness, safety, tolerability and positive influence on QoL in HD patients.

## 1. Introduction

Hemorrhoidal disease (HD) refers to the symptomatic manifestation of hemorrhoids, which are normal vascular structures located in the anal canal. While “hemorrhoids” describe a physiological condition, HD indicates a pathological state characterized by symptoms such as bleeding, prolapse, pain, perianal discomfort, anal leakage, perianal itching and burning [1]. Clinical manifestations of HD seem to occur mostly after hemorrhoidal nodules have begun their sliding down process [2]. Epidemiological data suggest that HD affects a significant portion of the adult population, with prevalence estimates ranging from 4% to 41% [3,4,5,6] depending on the population studied and the diagnostic criteria applied; despite its frequency, HD remains underdiagnosed due to patient reluctance to seek medical attention [7].

The pathogenesis of HD is multifactorial, involving mechanical, vascular, inflammatory and neoangiogenic mechanisms. Mechanically, the progressive weakening of the connective and muscular support of the anal cushions—often exacerbated by increased intra-abdominal pressure due to pregnancy, chronic constipation or prolonged straining—leads to their downward displacement and prolapse [8]. Hemodynamically, venous stasis and congestion contribute to vascular engorgement. Inflammatory mediators and oxidative stress further aggravate mucosal irritation. Recent studies have also highlighted the role of neoangiogenesis, with the formation of fragile, hyperpermeable vessels that may explain the frequent bleeding and chronic inflammation observed in symptomatic patients [8,9].

These mechanisms act synergistically and vary in prominence depending on individual patient characteristics. HD is classified anatomically as internal or external, depending on the location relative to the dentate line; external HD is clinically classified according to Goligher’s grading system (Grades I–IV), which reflects the degree of prolapse and reducibility [5,6,10].

In the clinical management of HD, it is essential to evaluate the degree and severity of the disease, its impact on quality of life (QoL), the intensity of symptoms, the likelihood of response to therapy, and the patient’s personal preferences. Treatments are generally grouped into two categories: supportive and interventional. Supportive measures consist of a high-fiber diet, fiber supplementation, increased water intake, warm water (sitz) baths, and stool softeners [11,12,13]. Current management of external and grade I and II HD is based on dietary and lifestyle modification combined with medical therapy or on interventional non-surgical procedures [2,14,15]. Topical medications available often contain either a combination of local anesthetics with anti-inflammatories or muscle relaxants, targeting single specific mechanisms involved in symptom onset [14].

Other medical therapeutic options that have been employed in the management of HD include flavonoids [15,16,17,18,19,20], calcium dobesilate [21], plant-derived substances [22] and essential oils (EOs) [23,24].

Indeed, plant-based products have shown promising results in the management of hemorrhoidal disease, particularly in alleviating symptoms such as pain, bleeding and itching. Their anti-inflammatory and antioxidant properties support their use as effective and well-tolerated alternatives to conventional therapies.

NeoFitoroid BioOintment (Aboca S.p.a.—Sansepolcro, Arezzo—Italy) [25] is a substance-based medical device (SBMD) indicated for topical treatment of HD, both internal and external.

A recent prospective interventional study evaluated the effect of this SBMD in decreasing the symptoms and signs of HD, showing the product as a valid non-pharmacological treatment option for the clinical management of grade I and II HD. In that study, data highlights how the unique combination of natural substances appears to target multiple pathophysiological mechanisms involved in the onset and severity of HD and its clinical manifestations [26]. In this context, the present research survey was conducted using an electronic platform to gather clinical data on the safety and effectiveness of the product in a real-word (RW) setting.

Continuous clinical evaluation throughout the life of products is required by the new EU Medical Devices Regulation (EU) 2017/745, which establishes updated criteria for the authorization, classification and post-market surveillance of medical devices [27]. This large-scale research aimed to assess the perceived efficacy, safety and usage patterns of the product among patients, physicians and pharmacists.

## 2. Materials and Methods

### 2.1. Product

Neofitoroid BioOinment [25] is a vegetal complex system containing Helydol^®^ (a freeze-dry extract of the *Helichrysum* lipophilic fraction) combined together with a pool of functional molecules, including an extract of Ruscus, a dry extract of Aloe Vera gel, an aqueous solution of Helichrysum, Hypericum oil, Shea butter, Jojoba oil, and essential oils of Melaleuca, Peppermint, and Cypress. This product is able to calm irritation, protect the mucosa and support its normalization through mucoadhesive, lubricant and antioxidant properties.

This SBMD does not contain corticosteroids or anesthetics, and its mechanism of action ensures that it does not irritate or dry out the mucosa. Indeed, it is indicated for the treatment of HD and its symptoms, such as pain, burning and anal and perianal itching. It can be used even in the case of anal fissures. This product can also be used in prevention and in all situations that can cause irritation and/or congestion of the anal and perianal region (constipation, diarrhea, unhealthy eating or lifestyle), and it is also suitable during pregnancy and breastfeeding.

### 2.2. Survey Research Design

A cross-sectional survey was conducted to assess the perceived efficacy and safety profiles of the product, involving three different cohorts: patients, physicians and pharmacists. All real-world clinical data (RWD) were collected through a structured GxP-compliant web platform (version 2.3.1.2), provided by Arithmos, an Italian ISO 27001–certified provider based in Verona. The platform is fully aligned with GDPR requirements, ensuring data protection and regulatory compliance [28]. The survey was carried out from 12 April 2021 to 3 November 2024, and it aimed to gather information through online questionnaires [28]. Patients accessed the platform via the website link or a QR code included on the product packaging. Product purchase was verified through the entry of a batch number and a unique code (both found on the packaging) prior to starting the online questionnaire. Likewise, physicians and pharmacists accessed the platform via a dedicated area for healthcare professionals on the manufacturer’s website. Participation was voluntary. Furthermore, physicians were directly recruited by the manufacturer’s scientific representatives. Clinical data were sampled using digital questionnaires developed by clinical experts in collaboration with the Department of Biomedicine, Surgery, and Dentistry at the University of Milan. These questionnaires were specifically designed for the surveyed cohort. Patients were also asked to share their experiences with the product, while physicians and pharmacists were asked to describe their patients’ experiences. This approach allowed for indirect validation of patient-reported data. To assess the accuracy of this survey, a preliminary reproducibility study was conducted before distributing the questionnaires to all three cohorts. This preliminary study also indirectly assessed the potential validity of the questionnaire research [29]. While the repeatability study was conducted for a similar product, the methodology and question structure were the same. Indeed, the repeatability of the results can be considered applicable to the current survey. The questionnaire’s main scope was focused on the following items:

- Effectiveness of the device in the treatment of HD (both internal and external) and their symptoms, such as anal and perianal pain, burning and itching.
- Evaluation of satisfaction levels associated with its use.
- Safety and tolerability.
- Quality of life (QoL).
- Compliance with the dosage suggested by the product.
- Concomitant treatments.
- Conditions for which the product was used/prescribed.
- Improvement and timing of problem resolution.
- Clarity of information.

**Figure 1:**
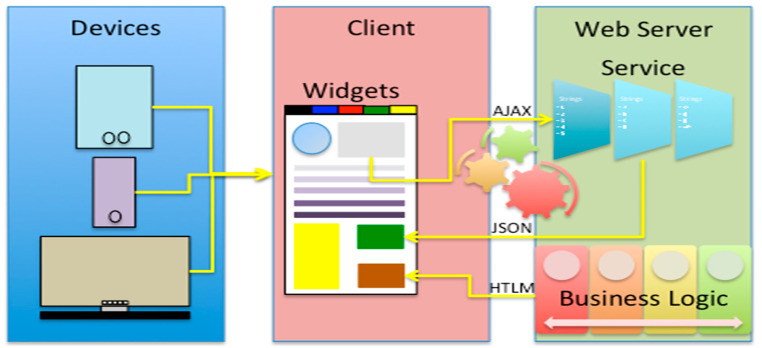
The real-world data (RWD) platform technology is implemented following a basic web architecture model. In this setup, a server—comprising web services and the business logic (i.e., the application layer)—communicates with the client by delivering HTML pages. Each page on the client side contains distinct components called *widgets*, which are interactive elements of the graphical user interface. These widgets can exchange data with the server by sending AJAX requests to the web.

At the end of the questionnaire, the reporting of any adverse event or interaction with the medical device could be submitted to the Medical Vigilance Department via a dedicated safety mailbox. The platform used for data collection is based on a web architecture that allows patients to interact with the system, as shown in Figure 1. Since 2021, each participant had confirmed having read and accepted the information provided, in compliance with Italian laws and the EU GDPR due to the non-anonymous nature of the data.

### 2.3. Sample Size

The sample size of this Real Word Evidence (RWE) survey is composed of the following cohorts: 1424 patients, 709 pharmacists and 485 physicians. This sample allows for estimation with the following error margins calculated for the populations included in the RWE survey—patients, 3%; pharmacists, 4%; and physicians, 4.4%—with a confidence interval of 95% and an estimated response rate to the questions of 50%. The calculations were performed using an online tool available at http://www.raosoft.com/samplesize.html (accessed on 4 November 2024). Descriptive analyses were performed for each question, with the findings presented as both absolute counts and percentages. For questions allowing single or multiple answers, the percentage reflects the share of responders who selected that option, unless specified otherwise.

### 2.4. Ordinal Likert Scale Data Trasformation and Quantitative Analysis

The transformation of ordinal Likert scale data and quantitative analysis described below were performed on Likert scale ratings obtained from participant cohorts through questions assessing the following items: impact on QoL, perceived effectiveness, safety and tolerability. To facilitate quantitative analysis, each response option, i.e., ‘*Excellent’*, ‘*Good’*, ‘*Fair’*, ‘*Adequate’* and ‘*Poor’*, was assigned a numerical value from 5 to 1, with the most favorable option (i.e., ‘*Excellent’*) corresponding to 5. These numerical values are referred to as Likert scale values (LSVs). Then, for each item and for each participant cohort, a global score (GS) was calculated according to Equation (1) to capture the overall cohort’s beliefs with respect to each individual item. The global score is obtained by summing the values that, for each response option presented, are obtained by multiplying the observed frequency of the response (answer frequency, AF) by its LSV. Therefore, the closer the GS is to 5—its maximum value—the higher the frequency of answers corresponding to the most favorable options is. Since these GSs are informed by the clinical RWD collected, they are referred to as RWE global scores. In the case of the patient cohort, two different questions concerning perceived effectiveness, both contained in the questionnaire, were considered to determine the two related global scores. To explore whether the answers could be provided randomly or not, a hypothetical parametric distribution (HPD) of the frequency of answers was assumed, and a second GS, namely the HPD global score, was calculated according to Equation (2). In this latter equation, all response options have the same frequency, assumed to be equal to 20%, so that the corresponding LSVs are multiplied by a fixed factor of 0.2. Therefore, the HPD global score is always equal to 3, whereas RWE global scores other than 3 reflect a deviation from randomness in the sense of a higher (score > 3) or lower (score < 3) observed frequency of answers with higher LSVs, i.e., answers corresponding to the more favorable options. The HPD global score is also the referral comparison term vs RWE global scores for the comparative statistics analyses. The ANOVA parametric test was used for testing statistical differences between the HPD and RWE global scores. *p* values <0.05 were considered significant.

(1) $$RWE=\sum_{N=1}^{5}(LSV_{n}\times AF_{n})$$

Equation (1): Global score (GS) based on observed Likert scale ratings. RWE = real-world evidence; LSV = Likert scale value; AF = answer frequency; N = Number of values for likert scale option.

(2) $$HPD=\sum_{N=1}^{5}(LSV_{n}\times 0.2_{n})$$

Equation (2): Global score based on hypothetical parametric distribution (HPD) of answers. LSV = Likert scale value; AF = answer frequency (0.2); N = numerical value for Likert scale options.

### 2.5. Descriptive Analysis

For each question, descriptive analyses were performed on the data from the ad hoc Likert scale. For both single- and multiple-response questions, the percentages shown represent the proportion of responders who chose that specific answer, unless indicated otherwise.

## 3. Results

### 3.1. Participants in the Survey

In total, 1424 patients, 709 pharmacists and 485 physicians spontaneously participated in the survey. Among the patients, 85.46% (N = 1217) were women, and their ages ranged from 17 to over 65 years old. The largest group within the patient cohort was represented by subjects aged between 31 and 50 years (N = 838). No anagraphic data were acquired from either the pharmacists or physicians.

### 3.2. Condition of Use

**Figure 2:**
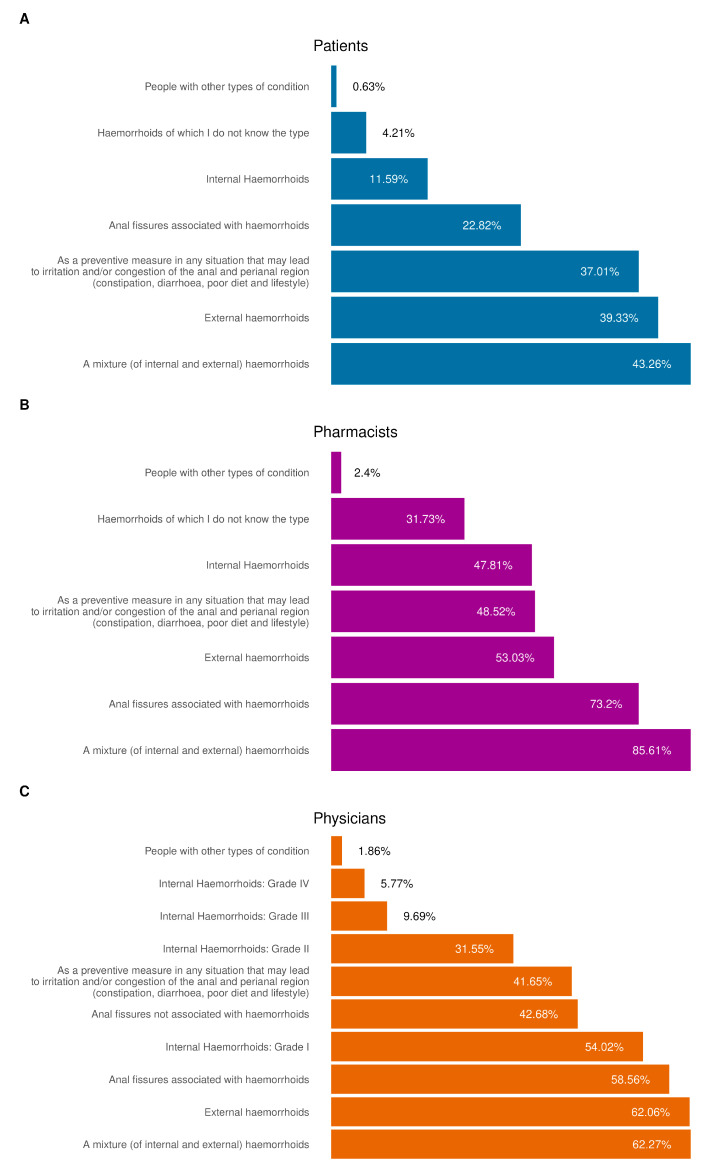
Conditions for which the product was used. (**A**) Distribution of conditions for which the product was used by patients; (**B**,**C**) distribution of conditions for which the product was recommended or prescribed by pharmacists and physicians. All results in the figure refer to multiple-response questions, with the percentages indicating the proportion of responders who selected each response.

The frequency and conditions of the treatment used across the cohorts is reported in Figure 2.

Based on the responses collected from patients, pharmacists and physicians, the most frequent condition across all three cohorts was a combination of internal and external hemorrhoids, particularly among patients (43.26%). External hemorrhoids were also commonly cited, with 39.33% of patients, 53.03% of pharmacists and 62.06% of physicians reporting this condition. Additionally, the collected data also highlighted a strong propensity toward self-medication within the cohort of patients.

### 3.3. Effectiveness

**Figure 3:**
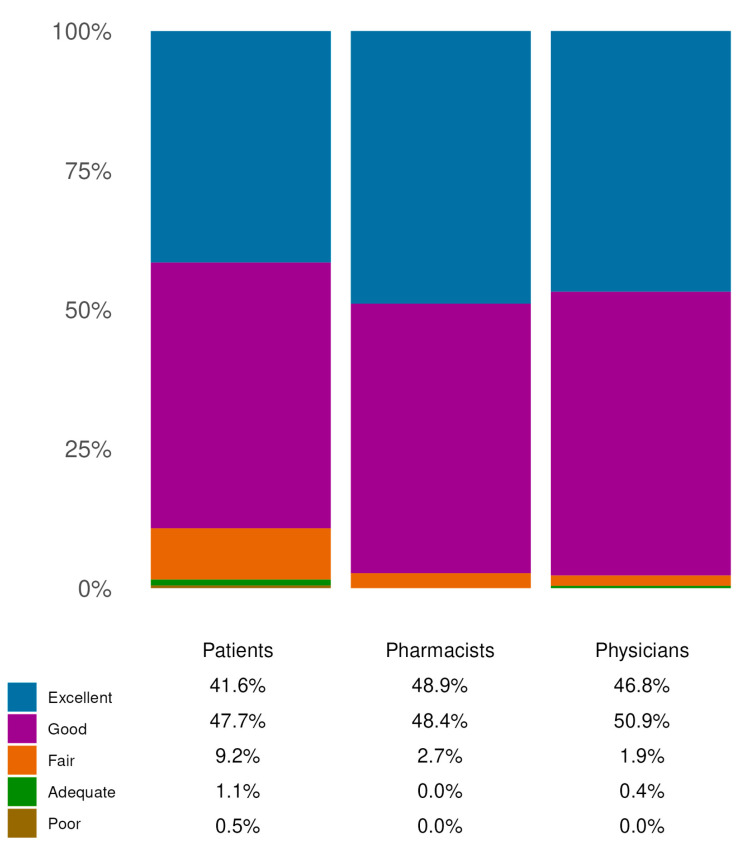
Effectiveness evaluation. Distribution of responses on the perceived effectiveness of the product across the three cohorts. The reported percentages are those of responders, within the indicated cohorts, who chose the indicated response options. The question on effectiveness was common to all three cohorts. All data refer to single-response questions.

The effectiveness of the SBMD was evaluated through specific questions addressed to patients, pharmacists and physicians. Patients were asked to express their level of satisfaction with the effectiveness of the product (Q1) and to report the extent to which their most common symptoms had improved following its use (Q2). Pharmacists and physicians were both asked to evaluate the degree of symptom improvement observed in patients using the product. Regarding the question on effectiveness common to all three cohorts, nearly 90% of patients (N = 1263) reported being highly satisfied with the product’s efficacy. Similarly, almost all physicians and pharmacists expressed satisfaction. Notably, among pharmacists and physicians, over 97% (N = 690 and N = 474, respectively) rated the efficacy as ‘*Good*’ or ‘*Excellent*’ (Figure 3).

### 3.4. Safety and Tolerability

**Figure 4:**
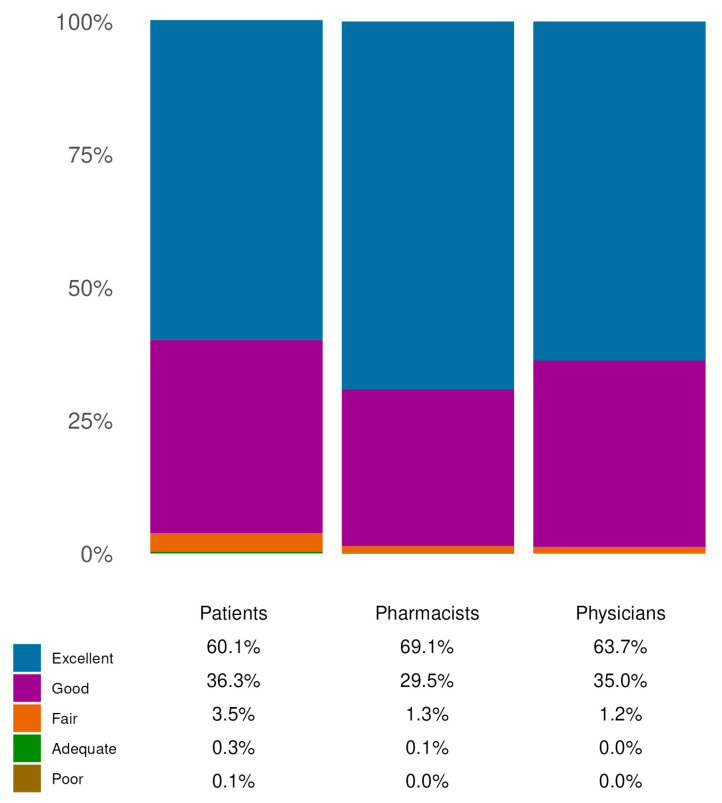
Safety and tolerability evaluation. The figure illustrates the assessment of safety and tolerability as reported by the three cohorts. The reported percentages are those of responders, within the indicated cohorts, who chose the indicated response options. The results in the figure are all extrapolated from single-choice questions.

Safety and tolerability were evaluated through a specific item included in the questionnaire and addressed to patients, pharmacists and physicians. These data suggest a favorable profile in terms of safety and tolerability. In particular, over 96% of patients (N = 1369) rated the product as ‘*good*’ or ‘*excellent*’. Pharmacists and physicians also reported high levels of satisfaction with the product’s safety profile, with 98.59% (N = 699) and 98.76% (N = 699) of responders, respectively, providing a positive evaluation (Figure 4). Only one patient reported a non-serious adverse effect (application-site pain), associated with device ineffectiveness. Potential systematic misuse of the product and off-label use were not reported as well.

### 3.5. Quality of Life

**Figure 5:**
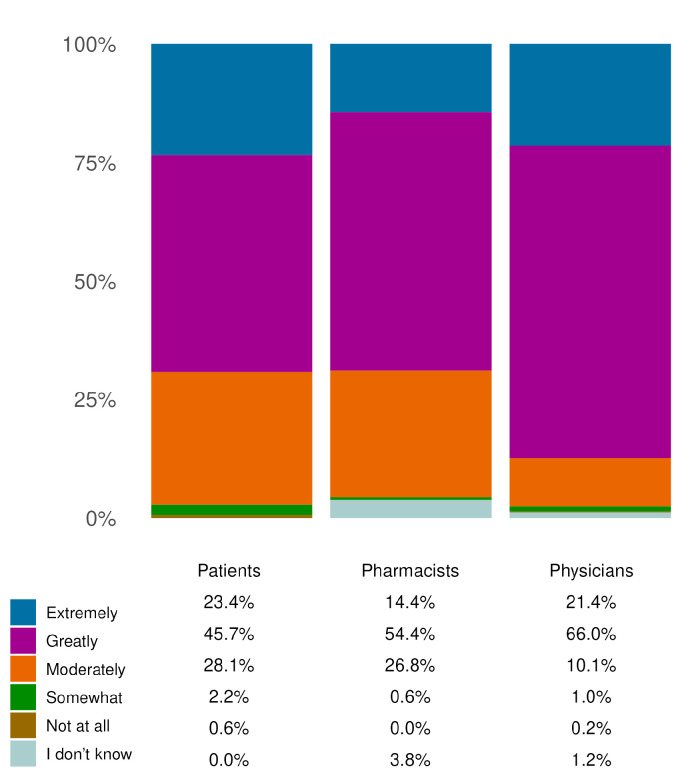
Effects on QoL. The improvement in QoL, as rated by the three cohorts, is indicated. The reported percentages are those of responders, within the indicated cohorts, who chose the indicated response options. The physician and pharmacist cohorts had the option to select “I don’t know”. All results in the figure refer to single-response questions.

The questionnaire also evaluated the improvement in the quality of life (QoL), considering aspects such as mood, social life, work, dietary freedom and physical activity. The results of this evaluation are presented in Figure 5. Most of the respondents in all cohorts rated QoL as ‘*Extremely*’ or ‘*Greatly*’ improved. The sum of the percentages of the answers indicating the two highest degrees of improvement in patients, pharmacists and physicians was equal to 69.11% (N = 978), 68.83% (N = 488) and 87.42% (N = 424), respectively.

**Figure 6:**
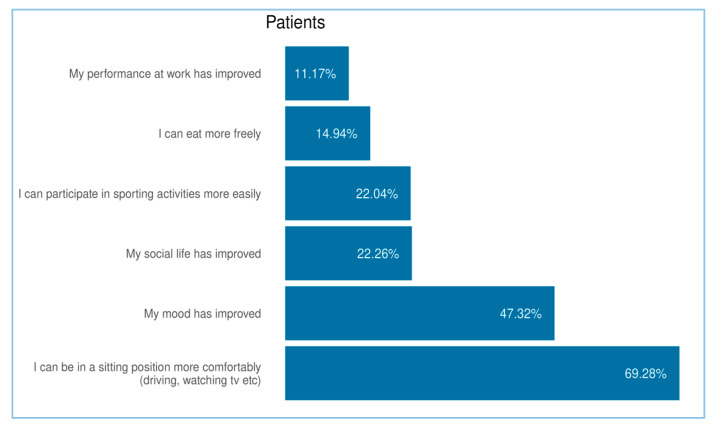
Analysis of the effects on patient’s QoL. The chart illustrates the percentage of patients who reported improvements in specific areas of daily life following the use of the product. All results in the figure refer to multiple-response questions, with the percentages indicating the proportion of responders who selected each response.

Within the patient cohort, the questionnaire also explored perceived improvements in specific aspects of daily life following the use of the product. The majority of responders (69.28%, N = 918) reported experiencing greater comfort while sitting. Additionally, 47.3% (N = 627) of patients indicated an improvement in mood. The other possible choices, including social life, sporting activity, dietary freedom and work performance, were indicated in similar percentages (from 11.17% to 22.26%), as reported in Figure 6.

### 3.6. Concomitant Treatments

**Figure 7:**
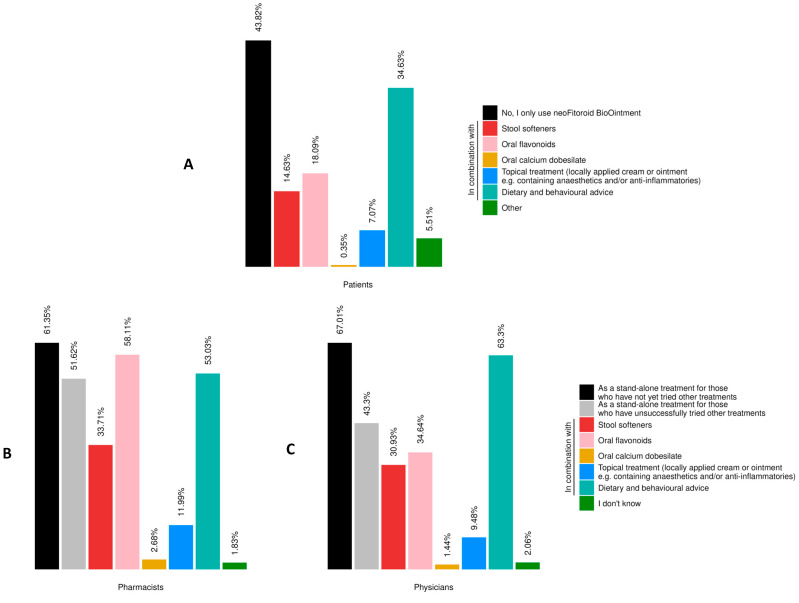
Products/interventions for the treatment of HD. (**A**) Distribution of different products/interventions used by patients. (**B**,**C**) Distribution of products/interventions recommended or prescribed by pharmacists and physicians. The physician and pharmacist cohorts had the option to select “I don’t know” (1.83% of pharmacists and 2.06% of physicians). All results in the figure refer to multiple-response questions, with the percentages indicating the proportion of responders who selected each response.

As part of the survey, patients, pharmacists and physicians were asked whether the product was used alone or in combination with other treatments (Figure 7). Among the patients, 43.82% reported using only the product and 34.63% reported using it in combination with dietary and behavioral advice. A smaller proportion (7.07%) reported combining it with other topical treatments, such as creams or ointments containing anesthetics and/or anti-inflammatory agents. Pharmacists and physicians also confirmed that the product was frequently used as a standalone treatment, particularly in patients who had previously experienced unsatisfactory outcomes with other treatments. Over half of the pharmacists (51.62%) and 43.30% of the physicians reported recommending or prescribing the product as a standalone option.

### 3.7. Quantitative Analyses

Quantitative analyses were performed to identify statistically significant differences in three key questionnaire traits: effectiveness, safety/tolerability and QoL. All three cohorts showed significant statistical differences when comparing the RWE vs. HPD data distributions. Notably, in all cohorts, responders more frequently selected higher Likert scale levels (Level 5: ‘*Extremely*’ and Level 4: ‘*Greatly*’).

Patients: Patients answered one question on QoL, two on effectiveness (Q1 and Q2) and one on safety and tolerability. The corresponding RWE global scores were 3.970, 4.290, 4.040 and 4.560, respectively. All scores were above 3, with a mean difference of 1.215. Compared to the HPD global score, these differences were statistically significant (ANOVA test, *p* = 0.0028, Table 1).

Pharmacists: Pharmacists responded to one question each on QoL, efficacy and safety/tolerability, reporting RWE global scores of 3.712, 4.460 and 4.380, respectively. All scores were above 3, with a mean difference of 1.184. Compared to the HPD global score, these differences were statistically significant (ANOVA test, *p* = 0.0378, Table 1).

Physicians: Clinicians answered the same three questions on QoL, efficacy and safety/tolerability, with RWE global scores of 4.037, 4.290 and 4.620, respectively. All scores exceeded 3, with a mean difference of 1.316. These differences were also statistically significant compared to the HPD global score (ANOVA test, *p* = 0.0161, Table 1).

**Figure 8:**
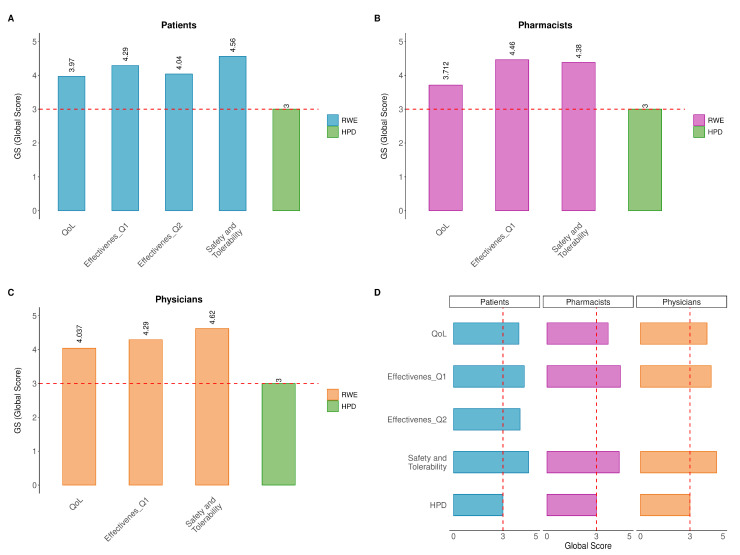
Quantification data. (**A**) Patient; (**B**) pharmacist; (**C**) physicians and (**D**) summary of RWE quantification by heatmap. Quantitative evaluation of effectiveness, safety/tolerability and quality of life (QoL) across three cohorts. The red dashed line indicates the expected global score (GS) value, equal to 3, in the random distribution. RWE: real-world evidence answers; HPD: hypothetical parametric distribution.

Collectively, these data indicate that all cohorts perceived QoL as highly satisfactory, with consistently elevated scores, highlighting a shared positive evaluation of QoL (Figure 8). Similarly, safety and tolerability were evaluated using the same Likert scale, and the results showed consistently high levels across all three cohorts. In fact, the mean of all questions for each cohort was well above 3 with at least one point more on average compared to the HPD global score. The overall results, with means and standard deviations, are reported in Table 1.

The combined frequency of the top two positive responses (‘*Extremely*’ and ‘*Greatly*’) indicated that the majority of participants perceived the product as both safe and well-tolerated. RWE and HPD data are both presented in Figure 8.

## 4. Discussion

Hemorrhoidal disease (HD) is often an underestimated pathology [2,30,31], sometimes because patients may find it difficult to discuss their symptoms with a physician [32]. Clinical management of HD depends on the degree of the disease, ranging from medical therapy for mild conditions (grades I and II) to surgical interventions for more severe conditions (grades III and IV) [5,33].

The most common therapeutic approach involves the use of calcium dobesilate [21] and natural compounds [22,23,24]. As acknowledged by the SICCR guidelines [2], self-management of HD is a commonly observed practice, which can represent a valuable approach for patient care. However, it is often challenging to accurately assess key data related to effectiveness, safety, tolerability and overall benefit for the patients [34,35,36]. In this context, RWE surveys represent a valuable framework for gathering data from real life, helping to monitor and better understand patient behaviors, treatment patterns and clinical outcomes in everyday settings and offering complementary insights that are often not captured in traditional clinical trials [28,29].

This survey presents the findings of a large-scale survey involving a total of 2618 participants from three distinct cohorts: patients, pharmacists and physicians. The primary objective of this survey research was to collect and represent RWD on the perceived efficacy, safety, tolerability and usage patterns of NeoFitoroid BioOintment in the treatment of internal and external hemorrhoids. Additionally, the survey aimed to assess patient satisfaction and QoL outcomes, compliance with the suggested dosage and the identification of any off-label uses and side effects. Data were collected through a newly validated, GDPR-compliant digital platform designed to support post-marketing surveillance and benefit–risk assessment [28] in full compliance with EU Regulation 2017/745 [27]. The online questionnaire was specifically developed and implemented for digital access, considering both healthcare professionals (HCPs, both pharmacists and medical doctors) and patients [29]. The data collected confirm that the RWE data are effective in capturing valuable insights, consistent with results reported in other studies [15].

The repeatability and validity of the questionnaires were assessed before the implementation of the survey. Since the structure of the questionnaires [28,29] has remained unchanged, it is reasonable to assume that the previously established repeatability can also be applied to the current version. Furthermore, a key strength of this survey is the simultaneous involvement of three interconnected cohorts, each actively involved in either using or recommending the product.

All three populations showed strong concordance and coherence on all items of the questionnaire. Notability, strong consistency regarding satisfaction with the product’s effectiveness was also observed, which was reported in the range of “*excellent/good*” across patients, pharmacists and physicians (Figure 3). Then, a large sample of patients reported great to extreme improvements in HD symptoms (78.69%), ultimately leading to an improvement in QoL. Remarkably, these positive perceptions were consistent across both HCP cohorts involved.

Similarly, the level of agreement regarding the safety and tolerability of the SBMD across the three cohorts was high. Indeed, over 90% of responders expressed positive evaluations (Figure 4). Furthermore, except for one case of application-site pain associated with device ineffectiveness, no potentially related adverse effects or interactions with other concomitant treatments were reported, supporting the product’s favorable profile.

In general, the questionnaire answers indicated the correct use and dosage of the product to protect the anorectal mucosa. Indeed, patients used the product to treat hemorrhoids, both internal and external, and anal fissures or as prevention for expected symptoms, such as anal and perianal pain or discomfort.

Regarding QoL, the consistent concentration of responses in the highest categories of the Likert scale (‘*Extremely*’ and ‘*Greatly*’) across all three cohorts suggests that the product is perceived as effective in improving key aspects of daily living, including mood, social interactions, work performance, dietary freedom and physical activity. This alignment between patient-reported outcomes and professional assessments reinforces the clinical relevance of the observed benefits in real-world setting.

A previous prospective interventional study by Podda et al. [26] evaluated the efficacy and tolerability of the product in 45 patients with grade I or II HD. After 10 days of treatment, a significant reduction in discomfort was observed, with the mean VAS score decreasing from 47.4 to 15.4 (*p* < 0.001). Similar improvements were reported for pain, itching and burning, with statistically significant reductions as early as day 3. Objective signs such as bleeding and anal leakage also decreased significantly (*p* < 0.001 and *p* < 0.05, respectively). Notably, 60% of patients with prolapse at baseline no longer showed signs of prolapse after treatment.

In addition, data on concomitant treatments indicate that the product is used both as a standalone therapy and as part of broader treatment strategies. This reflects its integration into multimodal regimens, often alongside dietary and behavioral advice or other topical and systemic treatments, and highlights its perceived value across different patient profiles and clinical scenarios.

In all cohorts, the minimum satisfaction level (89% in the patient cohort) was associated with a greater perceived positive impact of the treatment, while pharmacists and physicians evaluated the improvement as higher than 95% (Figure 3).

In recent years, there has been increasing interest in the use of natural compounds for the treatment of HD. This shift is driven by the need for safer, well-tolerated alternatives to conventional pharmacological or surgical interventions. Indeed, clinical evidence supports the efficacy of plant-based products in significantly reducing key symptoms of HD [5,12,13]. The present work reflects the growing interest, from both patients and the scientific community, in non-pharmacological therapeutic options. For patients, this interest is likely driven by multiple factors, including the perception of greater safety, concerns about prolonged medication use and partial satisfaction with currently available medical therapeutic options.

The study design and data collection methods present some limitations. Participation was partially self-selected and potentially biased, as completing the digital survey required both internet access and basic digital literacy. Furthermore, the assessment of patients’ quality of life was based on a limited set of indicators—specifically, perceived improvements in social life, physical activity, dietary freedom and work performance. These simplified measures were preferred over standard quality of life questionnaires to minimize the risk of low response rates due to survey length. As the data were collected through real-world surveys, they might be interpreted as reflecting the subjective experiences of end users (both patients and HCPs).

The statistical methods used to analyze the data revealed a quantitative output for them. In fact, the utilization of Likert scale ratings and the comparison of HPD vs. RWE global scores opened up the possibility of quantifying data from the survey related to RWD, through a specific test while also revealing the level of significance by generating a *p* value. When assessing the types of results from the responders, their answers highlighted “a causal distribution” instead of “a casual distribution” of data.

Overall, the product seems to fulfill the essential criteria for effective HD treatment: high safety features that allow for its use without specialist supervision, compatibility with other therapies, and efficacy in managing both internal and external hemorrhoids. The RWD collected provide valuable insights into the treatment’s clinical benefits and safety for the general population, supporting a robust benefit–risk assessment.

## 5. Conclusions

The survey, which included a broad sample of patients, pharmacists and physicians, provided consistent real-word insights into the product’s efficacy, safety, tolerability and impact on QoL. The analytical methods used confirmed the robustness of these outcomes. The reported improvements in symptoms and overall QoL support the product’s potential for self-use in the management of HD. However, further controlled studies are needed to confirm and strengthen these outcomes.

## Figures and Tables

**Table 1:** Summary statistics of RWE global scores (GSs) calculated according to Equation (1) for each cohort (Group) and for the indicated number (N) of items.

Group	Mean	SD	N
Patients	4.215	0.268	4
Pharmacists	4.184	0.292	3
Physicians	4.316	0.411	3

## Data Availability

The raw data supporting the conclusions of this article will be made available by the authors on request.
